# Long-Term In-Situ Monitoring and Analysis of Terrain in Gas Hydrate Trial Harvesting Area

**DOI:** 10.3390/s22041351

**Published:** 2022-02-10

**Authors:** Chen Cao, Hao Wang, Yongqiang Ge, Wei Wang, Jin Guo, Peng Zhou, Feng Gao, Jiawang Chen

**Affiliations:** 1 Ocean College, Zhejiang University, Zhoushan 316021, China; cc666@zju.edu.cn (C.C.); 12134002@zju.edu.cn (H.W.); ge_yongqiang@zju.edu.cn (Y.G.); wangwei92@zju.edu.cn (W.W.); 22034212@zju.edu.cn (J.G.); zp2010_ok@126.com (P.Z.); g_feng2010@163.com (F.G.); 2 Hainan Institute, Zhejiang University, Sanya 572025, China

**Keywords:** gas hydrate, terrain monitoring, MEMS-IMU sensor array, terrain reconstruct, long-term and in-situ device

## Abstract

With the increase in global energy demand, the exploration and development of natural gas hydrate in sea has become a research hotspot in recent years. However, the environmental problems that may be brought about by large-scale harvesting are still concerns. The terrain monitoring of the trial harvesting area can effectively prevent the geological disasters that may be caused by the development of hydrates. Therefore, we have developed a new terrain monitoring device, which can work in the deep sea for a long time. Firstly, the structure of the sensor arrays and bus-type control system of the device are introduced. Secondly, an arc model with an interpolation method is used for reconstruction of the monitored terrain. Thirdly, after the accuracy of the sensing arrays are verified in laboratory, the device was placed in the Shenhu area of the South China Sea for more than 6 months of in-situ monitoring. Finally, we analyzed the data and concluded that the terrain of the monitored area was relatively flat, where the maximum subsidence was 12.3 cm and the maximum uplift was 2.75 cm.

## 1. Introduction

**Figure 1:**
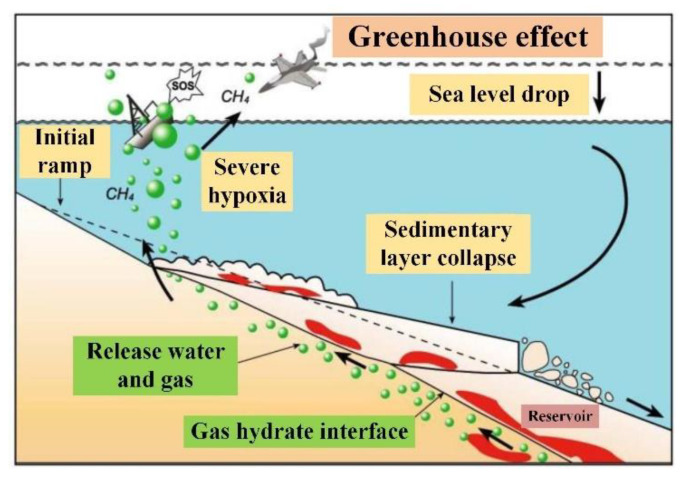
Decomposition of gas hydrates causes landslides [13].

Gas hydrate is an ice-like crystalline substance formed by natural gas and water under high pressure and low temperature, which produces minimal harmful pollutants after combustion. In particular, the carcinogenic substances such as SO_2_ produced are two orders of magnitude lower than those of crude oil or coal combustion, so gas hydrate is considered as a “green energy” [1,2,3]. The total resource of gas hydrate in the world is huge, where its organic carbon content is estimated to be about twice the total known coal, oil and natural gas in the world, which can meet human needs for the next thousand years [4,5]. While natural gas hydrate brings new energy prospects to humankind, it also poses severe challenges to the living environment. Gas hydrates consolidating in seafloor sediments release gases such as methane once environmental conditions change [6,7]. The consequence is uneven distribution of stratum bearing capacity, greatly reducing mechanical properties and softening the seafloor, which will cause large-scale earthquakes and landslides, destroying engineering facilities, such as submarine cables and offshore oil drilling platforms [6,8,9,10]. Figure 1 shows that the decomposition of submarine gas hydrate may lead to the destruction of the original stability zone, causing geological hazards. The famous Storrega landslide in Norway and the Cape Fear landslide in the United States are all related to the decomposition of gas hydrates [11,12]. Therefore, it is necessary to monitor the surrounding environment in the process of gas hydrate exploitation.

**Figure 2:**
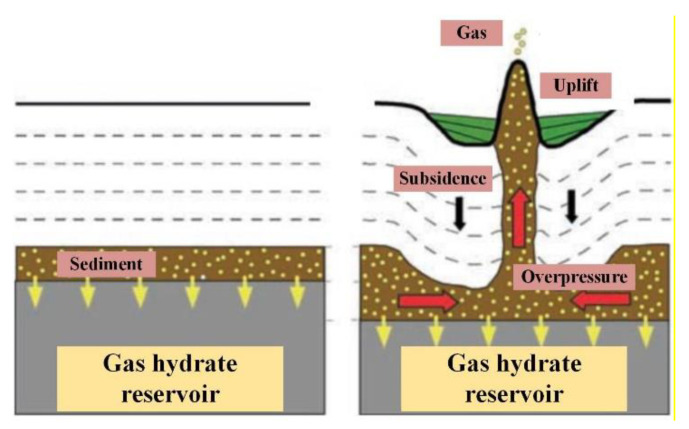
Relationship between gas hydrate decomposition and seafloor terrain subsidence [21].

Figure 2 shows the relationship between seafloor terrain subsidence and gas hydrate decomposition, while the velocity of subsidence or uplift directly reflects the stability of the stratum. In 2013, Japanese scholars used quartz pressure sensors to monitor the subsidence of a certain point during hydrate test harvesting [14,15]. The United States also established a hydrate seafloor observatory based on hydrophone arrays, temperature sensors and three-component accelerometers in 2015 [16,17]. In 2020, Chinese scholars set up long-term monitoring submersible markers for the seafloor environment during the hydrate test production, and the terrain subsidence of the wellhead was observed through an ROV (Remote Operated Vehicle) [18,19,20]. However, on the one hand, the monitoring of hydrate test harvesting areas is still mainly at a single point rather than a region, which may lead to the loss of key information. On the other hand, it is effective to observe terrain changes directly by ROVs, but the cost of using it is too high. Furthermore, the terrain subsidence (uplift) cannot be quantified.

The underwater terrain is measured mainly according to the relative relationship between the terrain and the station or sensor (distance, angle and coordinate difference, etc.), generally based on acoustic equipment [9,22]. Among them, single-beam echo sounders and multi-beam echo sounders mounted on scientific research vessels, ROV or AUV (Autonomous Underwater Vehicle) are widely used [23,24,25,26]. However, acoustic bathymetry is difficult to achieve accurate monitoring (with centimeter-level or higher accuracy requirements) [27]. In addition, the acoustics method cannot perform continuous monitoring for a long time, most of which are periodic short-term inspections [28,29]. Others, like satellite remote sensing, can calculate the depth of underwater terrain by obtaining the geoid height data, but it is difficult to penetrate the thick water to observe the seafloor [30]. Meanwhile, the submarine observation network has the advantages of being long-term, dynamic and real-time, which can be used for underwater terrain monitoring with a variety of sensors. Since the observation network is immobile, the monitoring area is relatively fixed, while expanding the monitoring range requires higher networking and maintenance costs [31,32,33]. Therefore, we consider developing a long-term in-situ monitoring device based on MEMS-IMU.

MEMS (micro-electro-mechanical systems) is a combination of microfabrication and IC (integrated circuit) technology to integrate microstructures, micro-sensors, micro-actuators, micro-control processing circuits, interfaces, communications and power supplies on one or more chips with feature sizes ranging from 10^−6^ to 10^−3^ m. It has the advantages of small size, light weight, low cost, low power consumption, high reliability and intelligence, etc. [9,34,35]. IMU (inertial measurement unit) is the sensor that mainly measures acceleration and rotation, generally consisting of an accelerometer and a gyroscope. It has the characteristics of autonomy and concealment, making it suitable for attitude measurement and navigation [36,37,38]. MEMS-IMU has the advantages of both, so it has been widely used in recent years. In environmental monitoring, a single MEMS-IMU has been purchased for landslide monitoring [39]. Some products with waterproofing, such as SAA of MEASURAND, are based on sensor arrays to monitor cable shape. However, they have strong limitations since the arrays are not fully flexible.

In this article, we have developed a new terrain monitoring device with four fully flexible MEMS-IMU sensor arrays, which can work in the deep sea for a long time. Then, we propose an arc model and an interpolation method to solve the problem of terrain reconstruction. The accuracy of the sensing array was verified through simulation experiments and the device was operated in the “Shenhu” area of the South China Sea for more than 6 months. Finally, we analyzed the data and concluded that the terrain of the monitored area was relatively flat, where the maximum subsidence was 12.3 cm and the maximum uplift was 2.75 cm.

## 2. Terrain Monitoring Device

**Figure 3:**
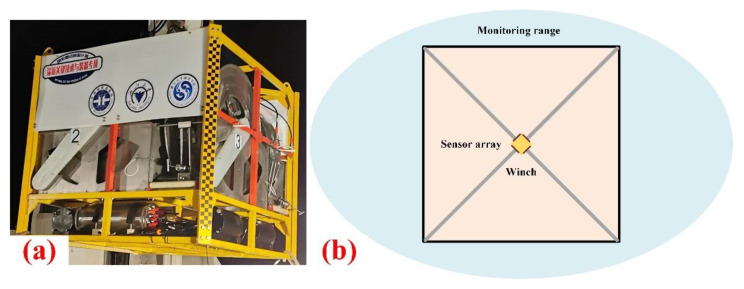
(**a**) Underwater winch; (**b**) Monitoring range.

The main body of the device is an underwater winch, which is mainly composed of four sensing arrays with control and auxiliary systems. When reaching the operating area, the winch is placed on the seafloor surface by the vessel. Then the manipulator of the ROV drags the four sensing arrays perpendicular to each other out of the specified length. Four arrays form the shape of an “X” as the diagonal to monitor a square plane, as shown in Figure 3.

### 2.1. Sensor Array

**Figure 4:**
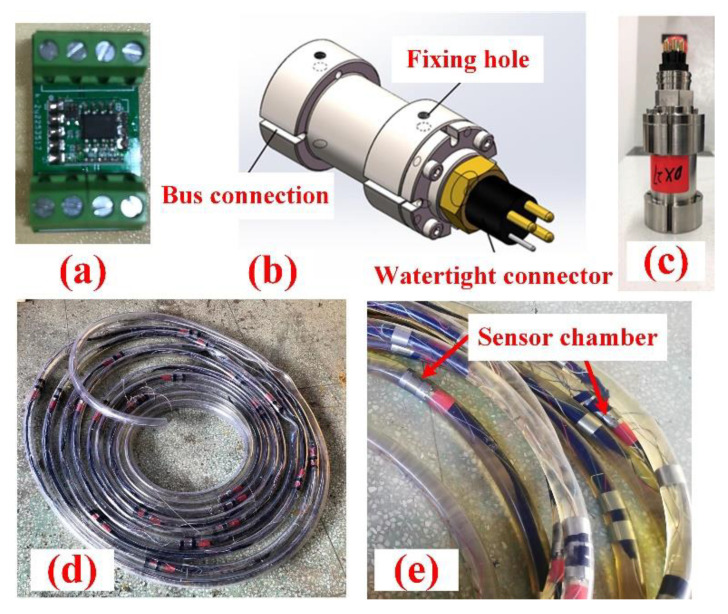
(**a**) MEMS-IMU; (**b**) Sensor chamber model; (**c**) Physical objects; (**d**) Oil-filled cable; (**e**) Sensor chambers in cable.

Each sensor array contains 21 MEMS-IMUs with a 1 m spacing. The total length of the array is 21 m, which can realize the monitoring range of about 30 × 30 m^2^ area. Since MEMS-IMU itself is not pressure-resistant, considering that gas hydrates are generally stored in the deep sea, we designed a small chamber to withstand pressure, as shown in Figure 4a–c. The main parameters of the MEMS sensor used are listed in Table 1.

In order to reduce deployment and retrieval resistance, sensor arrays are designed as oil-filled cables, which also make it flexible to better reflect terrain changes. In the meantime, oil can well isolate water to protect the chamber from corrosion as well as prevent short circuits. The structure of oil-filled cable is shown in Figure 4c,d.

### 2.2. Control System

**Figure 5:**
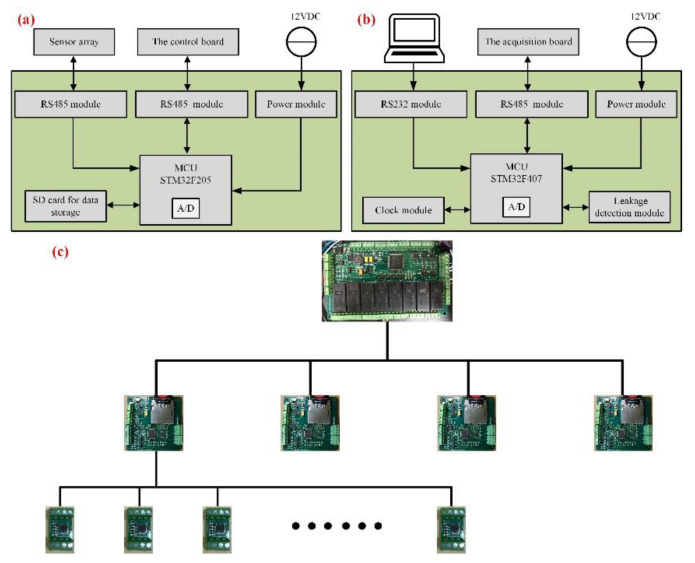
(**a**) “The acquisition board”; (**b**) “The control board”; (**c**) Control system structure.

The control system of the device is multi-level realized by the self-made PCB (printed circuit board) “the acquisition board” and “the control board”, as shown in Figure 5. Under the premise of long-distance communication, RS485 bus with multiple nodes is the first choice.

In our device, four “acquisition boards” supply power to the four sensor arrays, which can collect data from sensors with different physical addresses through an inquiry model. In this way, data loss due to bus congestion will be avoided and the synchronization of acquisition will be guaranteed. All sensors are connected on the bus to prevent the failure of a single sensor from affecting the array.

The four “acquisition boards” also have different physical addresses, which can communicate with the “control board” in term. The control board with leak detection can obtain the voltage and signal of the acquisition board to judge whether they are working normally. Furthermore, multiple relays on the control board can turn on and off the power of the acquisition board, respectively, which can reduce battery power consumption during long-term work through periodically waking up. Similarly, the acquisition boards and control board also have compartments for encapsulation and protection.

### 2.3. Remote Diagnosis Technology

**Figure 6:**
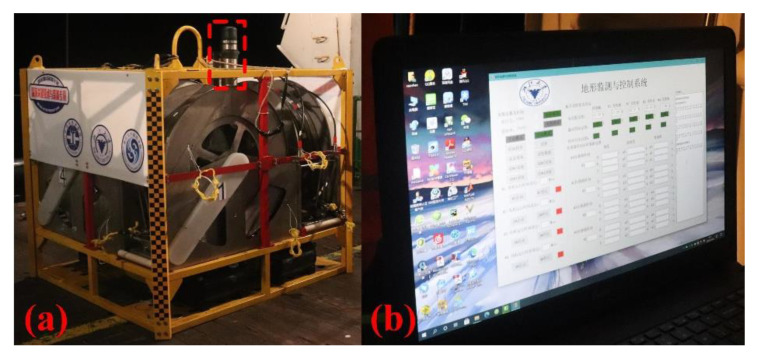
(**a**) Acoustic communication; (**b**) Software on host computer (normal status).

In the process of long-term in-situ work, we need to confirm the status of the device. It is difficult when the device is placed on the seafloor. Therefore, we are equipped with a 6000 m underwater acoustic communication on the top. When the vessel passes by the area, we can use software on a host computer to establish communication with the device to obtain information, as shown in Figure 6.

### 2.4. Power Supply

The device needs to operate in-situ on the seafloor for a long time, so sufficient battery power is necessary. Combined with the periodic wake-up working mode in Section 3.2, the battery power should consider two aspects of work and sleep consumption. The calculation formula is as follows:

(1) $$Q_{t}=I_{w}\times T_{w}+I_{s}\times T_{s}$$

where $Q_{t}$ is the overall power demand of the system; $I_{w}$ is the system working current; $T_{w}$ is the total system working time; $I_{s}$ is the system sleep current; $T_{s}$ is the total system sleep time.

In order to increase the stability of the system, we apply five batteries for power supply, four of which are used for the acquisition board, and the other used for the control board. The normal current of the acquisition board is 0.25 A, while it does not work when the system is sleeping. If the sensing array is operated for 10 min per day, the total power required to work continuously for six months is:

(2) $$Q_{t1}=I_{w1}\times T_{w1}=0.25\times 10/60\times 180=7.5\mathrm{Ah}$$

Considering the possibility of battery power loss at low temperature, we choose a battery of 12 V 15 Ah.

As for the control board, the current required is 0.02 A when sleeping, while it is 1.8 A at work. Accordingly, we can roughly calculate the required power:

(3) $$Q_{t2}=I_{w2}\times T_{w2}+I_{s2}\times T_{s2}=2\times 10/60\times 180+0.02\times 24\times 180=146.4\mathrm{Ah}$$

Therefore, a battery of 12 V 200 Ah is installed in the chamber for power supply.

## 3. Terrain Reconstruction Method

**Figure 7:**
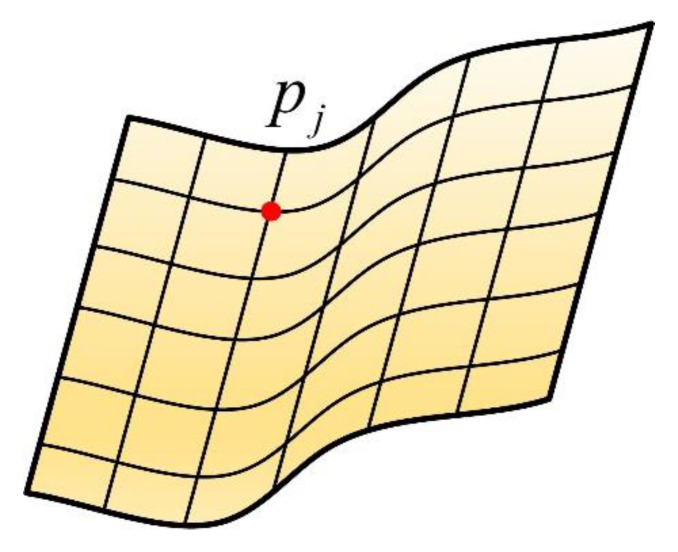
Surface to be solved.

In this research, we mainly focus on the subsidence and uplift of the terrain. Then the shape of the monitoring area can be represented by a surface, and the coordinates of each point should be solved, as shown in Figure 7.

Thus, we express the terrain by a set as follows:

(4) $$\Omega =\left\{p_{j}|j=1,2,\ldots ,n\right\}$$

where $p_{j}$ is a random point on the surface.

### 3.1. Pitch Angle

**Figure 8:**
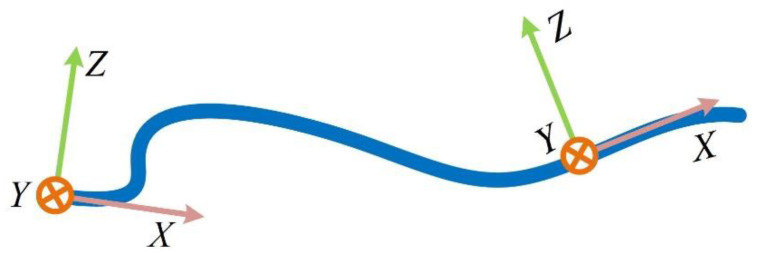
MEMS-IMU and array direction.

When designing, the “*X*-axis” direction of the MEMS-IMU coordinate system is consistent with the array, as shown in Figure 8.

On this condition, the fluctuation of the array is reflected through the angle around the “*Y*-axis”, that is, the pitch angle. According to the relationship between pitch angle and three-axis acceleration, we have:

(5) $$\theta_{i}=\arcsin\left(\frac{a_{xi}}{\sqrt{a_{xi}^{2}+a_{yi}^{2}+a_{zi}^{2}}}\right)$$

where $\theta_{i}$ is the pitch angle of $p_{i}$, and $a_{xi}$, $a_{yi}$, $a_{zi}$ are measurements of the sensor.

### 3.2. Arc Model

**Figure 9:**
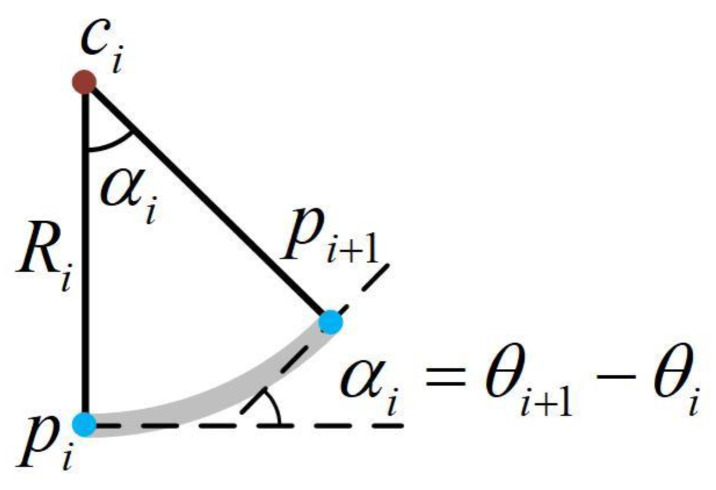
Arc model.

The subsidence and uplift occur on the “X-Z plane” for arrays, so dimensionality reduction can be performed. Since the cable is fully flexible, arcs can approximate the shape between the adjacent sensors. Therefore, we can calculate the relative position of the sensors through the central angle, as shown in Figure 9.

If the distance between the sensors is l, the position of $p_{i+1}$ relative to $p_{i}$ can be expressed as:

(6) $$p_{i+1}=\{\begin{array}{c} \left[\begin{array}{ccc} l & 0 & 0 \end{array}\right],\theta_{i+1}=\theta_{i}\\ {}[\begin{array}{ccc} \frac{l}{\left|\theta_{i+1}-\theta_{i}\right|}\sin(\theta_{i+1}-\theta_{i}) & 0 & \frac{l}{\left|\theta_{i+1}-\theta_{i}\right|}(1-\cos(\theta_{i+1}-\theta_{i})) \end{array}],else \end{array}$$

Taking the winch as a reference point, we can calculate the position of the sensors through rotation and iteration of the coordinate system, and thus reconstruct the shape of the cable.

**Figure 10:**
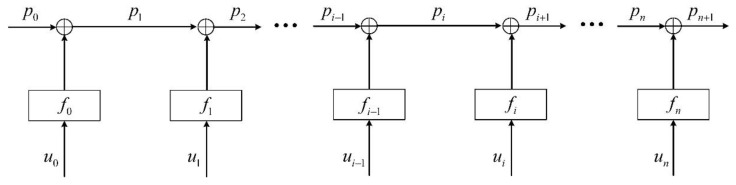
Recursion reconstruction model.

In general, the reconstruction algorithm obtains the coordinates of the curve by recursion, and its mathematical model can be shown in Figure 10.

Then the coordinates of point $p_{i+1}$ are solved as follows: the sum of the output after reconstructing the model f with the measurement value u of the sensor and the coordinate of the point $p_{i}$, namely:

(7) $$p_{i+1}=\sum_{m=0}^{i}f_{m}\left(u_{m}\right)+p_{0}$$

It can be seen from the above formula that the error of the $p_{i+1}$ mainly comes from the $p_{0}$, the model f and the input u. In order to facilitate the quantitative analysis of the model error, it is assumed that the errors of $p_{0}$ and u are 0. So, we have:

(8) $$\begin{array}{l} p_{i+1}=\sum_{m=0}^{i}f_{m}\left(u_{m}\right)+p_{0}+\sum_{m=0}^{i}\left(m+1\right)\Delta f_{i-m}\\ \Delta p_{i+1}=\sum_{m=0}^{i}\left(m+1\right)\Delta f_{i-m} \end{array}$$

where $\Delta f_{m}$ is the model error, which means that the general curve is equivalent to an arc in the calculation process. The model error $\Delta f_{m}$ is related to the specific shape of the sensor array, which can be positive, negative or zero for different shapes. If the mean is assumed to be 0, then the model errors of each segment can be considered as independent random processes, which belongs to an independent and identically distributed experiment with a mean of 0. According to the law of large numbers, we have:

(9) $$\underset{i\to \infty }{\lim}\Delta p_{i+1}=\underset{i\to \infty }{\lim}E\left[\sum_{m=0}^{i}\left(m+1\right)\Delta f_{i-m}\right]=\underset{i\to \infty }{\lim}\sum_{m=0}^{i}\left(m+1\right)\cdot E\left[\Delta f_{i-m}\right]=0$$

Therefore, when the distance between the sensors is constant, as the length of sensor array increases, the model error will converge, that is, the error of the $p_{i+1\left(i\to \infty \right)}$ will tend to 0; If the length of the sensor array is constant, the greater the number of sensors, the smaller the model error will be.

### 3.3. Neighborhood Subdivision Interpolation

**Figure 11:**
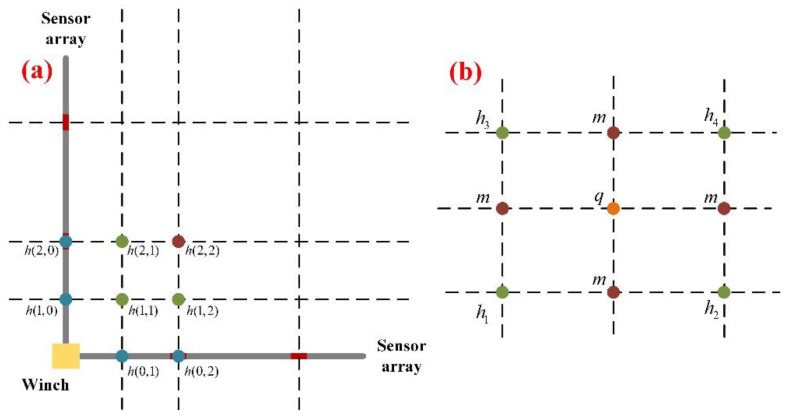
(**a**) Neighborhood interpolation; (**b**) Subdivision.

Based on the arc model, we can reconstruct the shape of the array, that is, the position of each point on the array is known. Then we still need to calculate the elevations of the four “triangle” areas in Figure 3. Thus, the “neighborhood interpolation” is applied in this case, the main idea of which is to give a certain weight to two adjacent points to calculate the fitting point, as shown in Figure 11a. Equation (10) gives the specific calculation method.

(10) $$\{\begin{array}{c} h(0,0)=0\\ i\geq 1,j\geq 1\\ i+j\leq m\\ h(i,j)=\frac{1}{2}(h(i-1,j)+h(i,j-1)) \end{array}$$

where m is the number of points divided by the sensor array, the larger the m, the smoother the reconstructed surface.

After obtaining the positions of the various points on the surface, we can further subdivide. New points can be inserted on each edge and in the middle of the mesh, connecting the new points to each other in a certain order to obtain a subdivided “quadrilateral”, as shown in Figure 11b. We define the weight of the center point as 0.25 and the weight of the midpoint on the edge as 0.5, that is:

(11) $$\begin{array}{l} m=0.5\times \left(h_{1}+h_{2}\right)\\ q=0.25\times \left(h_{1}+h_{2}+h_{3}+h_{4}\right) \end{array}$$

The above interpolation is very effective when the surface is continuous, that is, we consider that terrain have relevance. However, there may still be some errors in applications, while denser sensor arrays will improve the situation.

### 3.4. Solving for Time Series Subsidence (Uplift)

After solving the surface by the above method, we can record the shape at different moments, denoted as Ω(t). The amount of subsidence (uplift) can be obtained by calculating the difference in the shape of the surface at the time of $t_{1}$ and $t_{2}$, as shown below:

(12) $$D=\{d|d=\Omega (t_{2})-\Omega (t_{1}),t_{2}>t_{1}\}$$

Particularly, d>0 means uplift, while d<0 means subsidence.

**Figure 12:**
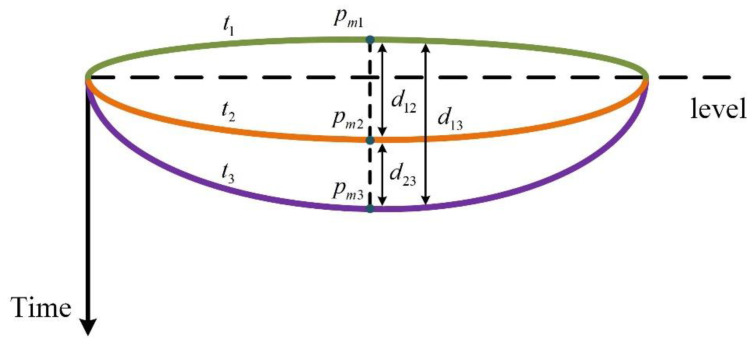
Solving for subsidence (uplift).

As shown in Figure 12, $p_{m1}$, $p_{m2}$ and $p_{m3}$ are three points on the surface at times $t_{1}$, $t_{2}$ and $t_{3}$, respectively. They have the same x and y coordinate values, and the difference in the z between the every two points is the subsidence, that is, $d_{12}$, $d_{13}$ and $d_{23}$ are the amount of subsidence from $t_{1}$ to $t_{2}$, $t_{2}$ to $t_{3}$ and $t_{1}$ to $t_{3}$.

## 4. Experiments

### 4.1. Subsidence (Uplift) Simulation Platform

**Figure 13:**
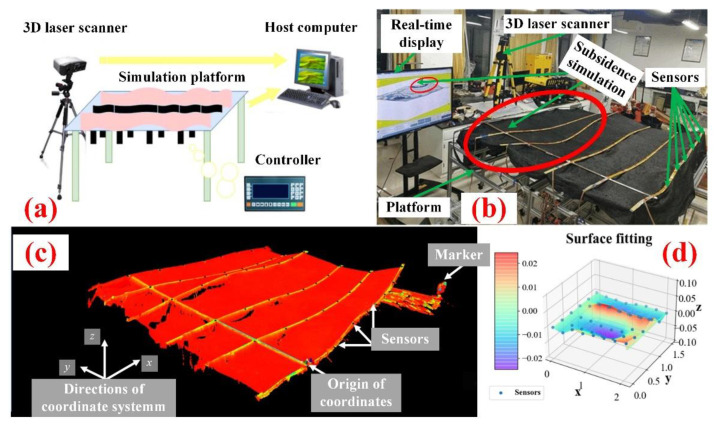
(**a**) Platform schematic; (**b**) Subsidence simulation experiment; (**c**) Surface in 3D laser scanner (**d**) Reconstructed surface.

In order to verify the accuracy of the sensor arrays and surface reconstruction, we built a subsidence simulation platform, as shown in the Figure 13a,b. Six sensor arrays are arranged on the black sunscreen with good plasticity, one end of which is fixed. There are seven sensors on each array with 30 cm spacing. The controller can drive the motor to change the height of the local position to simulate terrain subsidence or uplift. The high-precision 3D laser scanner is used to reflect the true shape of the sunscreen, which is compared with the surface reconstructed through sensor data, as shown in Figure 13c,d.

We construct three different surface shapes for reconstruction accuracy analysis, where the evaluation indicators are root mean square error (RMSE), maximum decision error (ME) and mean absolute error (MAE). Their definitions are as follows:

(13) $$\begin{array}{l} RMSE\left(X,h\right)=\sqrt{\frac{1}{n}\sum_{i=1}^{n}{\left(h(x_{i})-y_{i}\right)}^{2}}\\ ME(X,h)=\max(\left|h(x_{i})-y_{i}\right|)\\ MAE(X,h)=\frac{1}{n}\sum_{i=1}^{n}\left|h(x_{i})-y_{i}\right| \end{array}$$

We also count the extreme values of deformation and angle separately, as shown in Table 2.

From Table 2 we can draw the following conclusions:

(1) The difference between the reconstructed surface and the actual shape is small and the maximum absolute error of three shapes is only 2.34 cm;
(2) There is not an outlier where the reconstructed value is far from the true value;
(3) The deformation and angle have no obvious relationship with the reconstruction error.

### 4.2. In-Situ Monitoring in Harvesting Area

**Figure 14:**
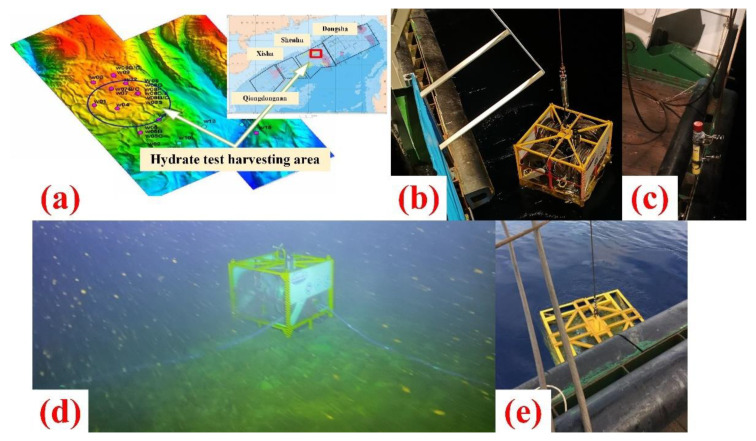
(**a**) “Shenhu” test harvesting area; (**b**) Device devolving; (**c**) Beacon (**d**) Sensor cables deployment; (**e**) Device recovering.

The device was carried on the 202007 voyage of the “Hangzhou Dizhi No. 6” scientific research vessel for in-situ monitoring. On the night of 19 November 2020, we arrived at the Shenhu test harvesting area in the South China Sea, where the water depth is about 1200 m, as shown in Figure 14a. After completing the final full inspection, we powered up the system. The ship-borne hoisting cable cooperates with the beacon (for device positioning) to deploy the device to the seafloor at a speed of not more than 50 m per minute, as shown in Figure 14b,c. Then we use the acoustic communication to inquire the status of the control system. After getting the feedback, we confirmed that the device is normal. Based on the location information recorded by the beacon, the “Haima-2” ROV used the front sonar to find the device. Afterwards, the manipulator of ROV pulled out the four sensing cables in turn, so that they were placed vertically against the seafloor surface, as shown in Figure 14d.

On 21 June 2021, we took “Haiyang Dizhi No. 6” again to recover the device, which was towed to the vessel by ROV, as shown in Figure 14e. After inspection, although there was some corrosion, the functions of the device were basically normal. The device had been deployed on the seafloor for 216 days so far. Meanwhile, the control system worked and collected one set of data every day as programmed. However, limited by the battery, the data of four acquisition boards did not match 216 groups, which were 205, 201, 206 and 193, respectively. That is, the device had completely collected terrain data for 193 days, and we focused on the changes in terrain during this period.

## 5. Data Analysis

**Figure 15:**
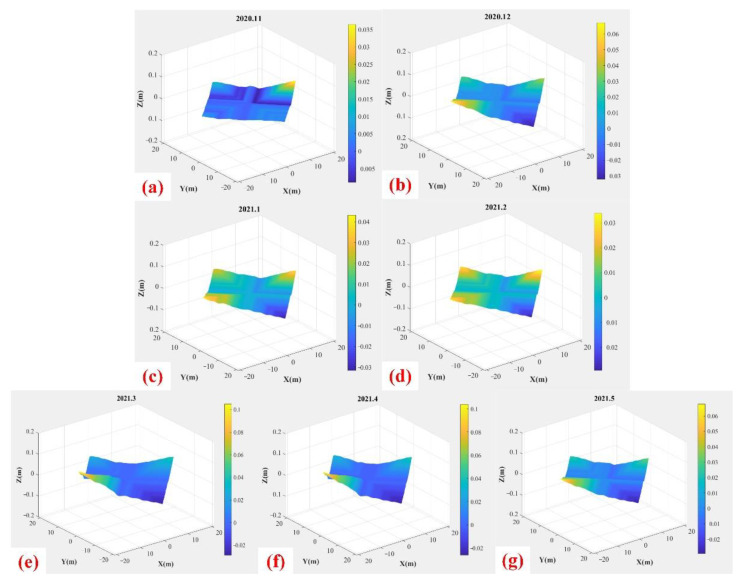
Terrain maps from November 2020 to May 2021.

The device collected a total of 193 sets of complete topographic data from 20 November 2020 to 29 May 2021. Then we reconstruct 193 terrain maps, some of which are shown in Figure 15 (25th of every month).

From the figure, it can be seen intuitively that the terrain changes little, which is reasonable since no harvesting was carried out during the monitoring process.

### 5.1. Amount and Velocity of Subsidence (Uplift)

The subsidence (uplift) of the terrain can be calculated using the method in Section 3.4. According to the interpolation in Section 3.3, we can infer that the largest subsidence (uplift) occurs on the sensing arrays. Therefore, we mainly focus on the monitoring area of the sensor arrays. For the special case of subsidence and then uplift at some locations, they are counted separately. Meanwhile, velocity is the amount of terrain change per unit time, which can be calculated as follow:

(14) $$v=\left|\frac{d}{t_{2}-t_{1}}\right|$$

Subsidence and uplift are not distinguished when calculating velocity. Taking into account the monitoring error of the device, the change we determined is defined as follows:

(1) The amount of subsidence (uplift) is more than 0.5 cm;
(2) Changes should persist for more than 3 days.

The result is shown in Table 3.

The data in the table show that the change in terrain is not large, but there is certain geological activity in this area, which may be caused by the hydrate phase change. In addition, the subsidence of the terrain is more obvious than the uplift, probably because the hydrate is decomposed into gas and lost from the soil. On the other hand, the velocity of the terrain change is small, which means that this area is relatively stable where geological disasters are unlikely to occur.

### 5.2. Terrain Amplitude-Frequency Characteristics

We extract data at equal intervals on the reconstructed terrain and perform a two-dimensional discrete Fourier transform (DFT) on it, the formula is:

(15) $$\begin{array}{l} F(u,v)=\sum_{x=0}^{M-1}\sum_{y=0}^{N-1}f(x,y)e^{-j2\pi (ux/M+vy/N)}\\ F(u,v)=\left|F(u,v)\right|e^{-j\phi (u,v)}\\ u=0,1,2,\ldots ,M-1\\ v=0,1,2,\ldots ,N-1 \end{array}$$

where M and N are the number of samples in the x and y, respectively. The frequency resolution of the terrain magnitude is inversely proportional to the spacing of the spatial sample points and the number of samples, as shown below:

(16) $$\{\begin{array}{c} \Delta u=\frac{1}{M\Delta x}\\ \Delta v=\frac{1}{N\Delta y} \end{array}$$

We sampled the terrain with equal intervals of 0.1 m in the x and y. The number of samples in both directions is 300, that is, the sampling frequency is:

(17) $$f_{s}=300/10m=30m^{-1}$$

According to Equation (16), we have:

(18) $$\{\begin{array}{c} \Delta u=0.033\\ \Delta v=0.033 \end{array}$$

**Figure 16:**
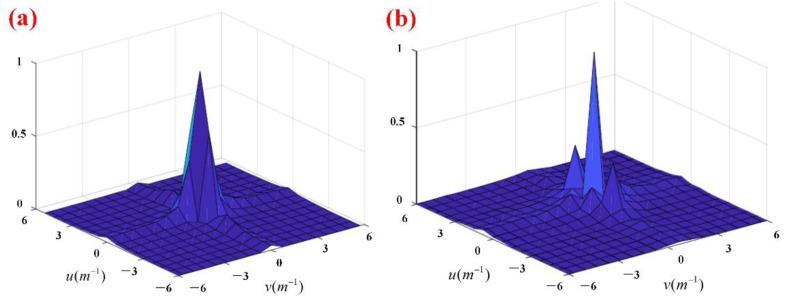
(**a**) Terrain amplitude spectral distribution in November 2020; (**b**) Terrain amplitude spectral distribution in May 2021 (local enlarged, normalization and centralization).

Figure 16 is the two-dimensional amplitude spectrum of the terrain. It can be seen from the figure that the dominant frequency of both in the u and v is 0, which means that the terrain of the monitoring area is flat. The conclusion has been verified in the previous exploration of the Shenhu sea area [40,41,42], and it is consistent with what was recorded by the ROV front camera in Figure 14d.

## 6. Conclusions

In this article, we have developed a new device for long-term in-situ terrain subsidence (uplift) monitoring of gas hydrate test harvesting areas, which is mainly composed of four watertight sensor cables with bus-type control system. Then we present an effective method for terrain subsidence (uplift) calculation. With a small amount of acceleration data from the MEMS-IMU sensors, the position of nodes in array can be solved by the arc model. On this basis, the terrain can be reconstructed through subdivision and interpolation. The simulation experiment in the laboratory verifies the accuracy of sensor array and reliability of the method. Afterwards, the device was placed in the Shenhu area of the South China Sea, which worked continuously for more than 6 months. Finally, we analyzed the data and concluded that the terrain in the monitored area was flat and changed slowly, where the maximum subsidence was 12.3 cm and the maximum uplift was 2.75 cm.

In summary, the scientific novelty of this research is listed as follow:

(1) We have designed a new device for long-term in-situ seafloor terrain monitoring that was proven to be reliable in the sea trial;
(2) We propose an arc model to calculate the shape of the sensor array and eliminate the possible interference caused by model errors;
(3) We introduce a novel method based on interpolation and subdivision to reconstruct the terrain of the monitored area.

In the future, we will further improve the performance of the device. On the one hand, we can expand the monitoring range and accuracy through increasing the number of sensing cables. On the other hand, the cruising ability of the device can be improved by equipping a larger capacity battery. Moreover, we plan to use the device for environmental monitoring during hydrate exploitation, which will be very helpful for disaster avoidance and subsequent analysis of soil mechanical properties.

## Figures and Tables

**Table 1:** The main parameters of the MEMS sensor.

Category	Index
Voltage	3.3–5 V
Current	<25 mA
Volume	15.24 mm × 15.24 mm × 2 mm
Measuring range	Acceleration ± 2 g; Angle ± 180°
Resolution	Acceleration 0.001 g; Angle 0.005°
Precision	Acceleration 0.01 g; Angle 0.01°

**Table 2:** Simulation experiment statistics.

	Shape 1	Shape 2	Shape 3
RMSE (cm)	1.04	0.78	0.95
ME (cm)	2.06	1.85	2.34
MAE (cm)	1.12	0.97	1.09
Maximum deformation (cm)	17.3	14.1	21.4
Minimum deformation (cm)	0.65	1.11	0.81
Maximum pitch angle (°)	32.22	28.03	45.96
Minimum pitch angle (°)	0.86	2.12	2.50

**Table 3:** Amount and velocity of subsidence and uplift.

	Array 1	Array 2	Array 3	Array 4	Overall
MES ^1^ (cm)	4.32	4.69	5.14	7.71	5.47
MS ^2^ (cm)	6.68	9.83	8.62	12.3	12.3
MEU ^3^ (cm)	1.12	0.97	0.84	0.53	0.87
MU ^4^ (cm)	2.75	1.68	1.36	0.79	2.75
MEV ^5^ (× 10^−2^ cm/day)	3.01	1.34	3.95	7.56	3.97
MV ^6^ (× 10^−2^ cm/day)	5.13	2.99	7.43	15.8	15.8

^1^ Mean subsidence; ^2^ Maximum subsidence; ^3^ Mean uplift; ^4^ Maximum uplift; ^5^ Mean velocity; ^6^ Maximum velocity.

## Data Availability

Not applicable.
